# Reversal of the Upward Trend of Obesity in Boys, but Not in Girls, in Spain

**DOI:** 10.3390/ijerph18041842

**Published:** 2021-02-14

**Authors:** Romana Albaladejo-Vicente, Rosa Villanueva-Orbaiz, David Carabantes-Alarcon, Juana Santos-Sancho, Rodrigo Jiménez-García, Enrique Regidor

**Affiliations:** 1 Department of Public Health & Maternal and Child Health, Faculty of Medicine, Universidad Complutense de Madrid, 28040 Madrid, Spain; ralbadal@med.ucm.es (R.A.-V.); dcaraban@ucm.es (D.C.-A.); juana.santos@med.ucm.es (J.S.-S.); rodrijim@ucm.es (R.J.-G.); eregidor@med.ucm.es (E.R.); 2 CIBER Epidemiología y Salud Pública (CIBERESP), Instituto de Salud Carlos III, 28029 Madrid, Spain

**Keywords:** prevention, diabetes, overweight, obesity, children, prevalence, surveys

## Abstract

(1) Background: To compare the prevalence of overweight and obesity in boys and girls and to estimate socioeconomic differences associated with obesity in Spain in 1997, 2007, and 2017. (2) Methods: Data were drawn from national health interview surveys. For each year of study, the prevalence of overweight and obesity was measured, and these results were compared by gender (boy/girl) and socioeconomic status (low/high education). (3) Results: The prevalence of overweight and obesity rose from 1997 to 2007 but then fell in 2017 in all subgroups except in girls aged 10 to 15 years. In this group, there was a steady increase in the prevalence of both overweight (1997, 14.6%; 2007, 17.7%; 2017, 19.6%) and obesity (1.1, 3.2, and 3.7%, respectively). The decrease in prevalence of overweight in both sexes and of obesity in boys, along with the increase in prevalence of obesity in girls, was of a higher magnitude in children whose parents had a lower educational level. (4) Conclusions: The apparent turnaround in the obesity epidemic in Spain should be interpreted with caution. Children’s body weight is influenced by both gender and socioeconomic status—considerations that should be kept in mind when designing health promotion interventions.

## 1. Introduction

Overweight and obesity are important health problems that incur immediate and negative consequences, including pediatric hypertension, abnormal glucose tolerance, and osteomuscular, neurological, pulmonary, gastrointestinal, and liver alterations [1,2]. Moreover, in the long term, childhood overweight and obesity increase the risk of chronic disease in adulthood, along with mental health disorders and social stigmatization [3]. From a health system perspective, childhood obesity and derived diseases lead to an increase in premature disability and mortality, and the associated treatments are responsible for the highest healthcare costs in children and young people [4].

The prevalence of overweight and obesity has been on the rise in recent decades, globally and in all age groups, including in children and adolescents. In the United States, childhood overweight has doubled in children and tripled in adolescents over the past 30 years [5].

However, since the turn of the century, different studies in high-income countries have shown that the prevalence of childhood overweight and obesity have stabilized. In one study in nine European countries and Australia, weight gain stalled between 1995 and 2008 [6], while other authors have reported similar findings in the UK, Ireland, and Sweden [3] as well as in China [7]. In Spain, the overall results for the most recent studies investigating trends in childhood overweight and obesity have also reported flattening rates for these indicators [8].

Other lines of contemporary research have studied the influence of socioeconomic factors on the risk of excess weight. In adults living in high-income countries, there is a clear, negative relationship between socioeconomic status (SES) and obesity [9]: the higher a person’s SES, the lower their weight. However, this relationship is less clear in children and adolescents [10,11,12]; in one multicenter study in Europe, heterogeneous relationships were observed between the parents’ SES and children’s body mass index (BMI) [9].

In childhood, the inverse relationship between obesity and SES was first reported by Stunkard et al. [13] in 1972; authors observed a six-fold higher prevalence of obesity in girls from families with low SES along with a lower-magnitude association in boys. Therefore, the aim of this study was to estimate the prevalence of overweight and obesity in boys and girls in Spain and to compare trends from 1995 to 2017. A secondary aim was to assess differences in overweight and obesity according to the parents’ educational level.

## 2. Materials and Methods

### 2.1. Study Design and Data Collection

This study used data from Spain’s national health surveys from 1995–1997, 2007, and 2017. Subjects were chosen by means of stratified multistage sampling: the first-stage units were census sections; the second-stage units, the household; the third-stage units, the selected people in the household (children and adolescents aged 5 to 15 years). The nonresponse rate in the interviews ranged from 4 to 10%.

### 2.2. Study Variables

BMI (kg/m^2^) was calculated according to the weight and height reported by parents or guardians. Overweight and obesity were defined according to Cole et al.’s 14 criteria for children and adolescents aged 2 to 18 years, which establish BMI cutoffs derived from the International Obesity Task Force recommendations [14] in adults (overweight: 25 kg/m^2^, obesity: 30 kg/m^2^). Nonresponse to items for weight and height were 32.1% in 1995–1997, 21.8% in 2007, and 10.6% in 2017.

The head of household’s educational level was used to measure SES [15]. The nonresponse rate for this variable ranged from 34.6% in 1997 to 10% in 2017. Responses were dichotomized as low educational level (up to 10th grade—the compulsory level of education in Spain for adolescents up to 16 years) and high educational level (educated beyond the minimum compulsory studies).

We excluded children with missing data for weight or height, which may have increased the risk of information bias if the participants with missing data were systematically different from those without. Additionally, in the analyses according to educational level, we have excluded children with missing data for this variable. However, there was no difference in the distribution by age and sex between those who answered and did not answer by weight and height, there was no relation between the measure of socioeconomic position used here and the nonresponse.

### 2.3. Statistical Methods

Separate analyses were performed for boys and girls. We first studied prevalence of overweight and obesity in children aged 5 to 15 years before stratifying the results by age: 5 to 9 years and 10 to 15 years. Prevalence of overweight and obesity was calculated by gender and age group, and results were expressed with their 95% confidence interval (CI). Trends in prevalence were determined by analyzing results from the three surveys, using the logistic regression models adjusted by age.

The relationship between overweight/obesity in boys and girls and their parents’ educational level was expressed as the odds ratio (OR) and 95% CI, adjusted for age and using the high-education group as the reference.

## 3. Results

**Figure 1:**
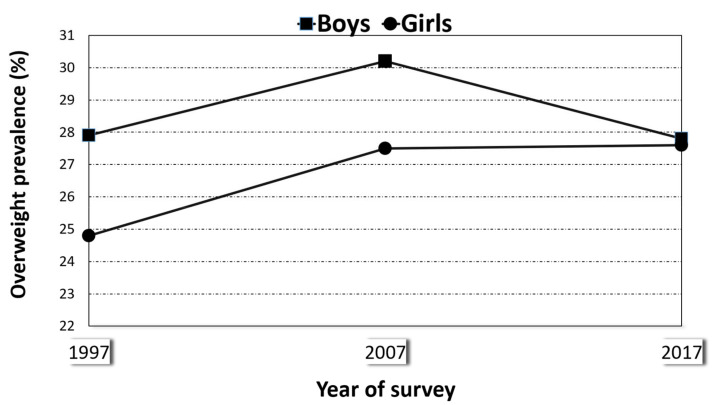
Prevalence of overweight in boys and girls aged 5 to 15 years, Spain (1997, 2007, and 2017).

**Figure 2:**
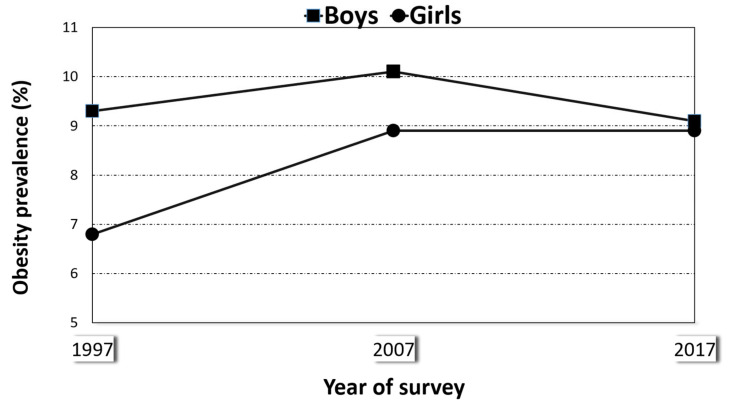
Prevalence of obesity in boys and girls aged 5 to 15 years, Spain (1997, 2007, and 2017).

Figure 1 shows the evolution of overweight from 1995–1997 to 2017 in all children aged 5 to 15 years. In boys, the prevalence rose from the first to the second survey and fell thereafter, while in girls, the initial increase (in 2007) was followed by a period of stability (to 2017). Figure 2 shows the results for obesity, which followed a similar pattern: prevalence rose and then fell in boys, and it rose and then stayed flat in girls.

In boys in the younger age group, there was an 11% decrease in prevalence for both overweight and obesity along the whole study period (Table 1). In girls of this age group, overweight and obesity rose modestly from 1995–1997 to 2007 and then began to taper in 2017. The relative difference between the first and third surveys was a 1.4% increase in overweight and a 14% increase in obesity (Table 1). These changes in prevalence between another year were not statistically significant (*p* > 0.05).

In boys aged 10 to 15 years (Table 2), the prevalence of overweight rose notably in 2007 and descended very slightly in 2017, for a net 17% increase relative to the first survey. The prevalence of obesity rose by more than 40% between the first and second surveys, and although the trend changed direction in 2017, there were still 36.1% more obese boys compared to the 1995–1997 survey. Girls in the older age group also saw a pronounced increase (34%) in prevalence of overweight from 1995–1997 to 2017. Obesity rates also jumped, tripling in prevalence from 1.1% in the 1995–1997 survey to 3.7% in the 2017 survey (Table 2). These changes in prevalence across time were not statistically significant (*p* > 0.05).

The mean BMI reflects the same trend as overweight and obesity. Figures S1 and S2 show that the mean BMI decreased in 2017 compared to 2007, while the magnitude of the mean between 1997 and 2007 was similar or somewhat higher in 2007.

Table 3 presents the ORs for overweight and obesity in the total sample (5 to 15 years old), by gender and parents’ educational level and adjusted for age group. Number of subjects analyzed were: 1783 in 1997, 4616 in 2007, and 3570 in 2017. Low educational level in parents increased their sons’ risk of being overweight for all three survey periods. In girls, this association was not clear in the 1995–1997 data, but in both 2007 and 2017, it was stronger than that in boys.

Regarding the association between parental education and childhood obesity in boys (Table 3), the first survey shows an increased risk bordering on statistical significance, and in the second, the relationship is unequivocal. By the 2017 survey, however, the magnitude of the association weakened once again. In girls, no clear relationship between parents’ education and obesity existed in the 1995–1997 data; the 2007 data were suggestive of an increased risk, and by 2017, parents’ low educational level was associated with a two-fold increase in their daughters’ risk of being obese.

## 4. Discussion

This study investigates trends in prevalence for overweight and obesity in children (aged 5 to 9 years) and adolescents (aged 10 to 15 years) over a period of 20 years, using data from national health surveys undertaken in 1995–1997, 2007, and 2017. We observed a stronger tendency toward stabilization in boys’ BMI, especially with regard to overweight in 10- to 15-year-olds. In contrast, this trend was apparent only in girls aged 5 to 9 years, whereas in older girls, the prevalence of overweight and particularly obesity increased. 

Assessment of how these trends are related to SES revealed a stabilization in differences for overweight (in both boys and girls) and in obesity (in boys). In girls, socioeconomic differences related to obesity were exacerbated in the most recent survey (2017). 

Data on weight and height were those reported by the head of household in this study, as in other research in Spain [16,17] and internationally [4,12,18,19]. Validation studies for parent-reported anthropometric measures in children aged 6 to 8 years in Spain have shown a sensitivity of 78% and a specificity of 96% [16]. A similar study in Germany reported that sensitivity ranges from 78 to 85% and specificity from 97 to 99% [20].

Our results also show how factors such as age, gender, and SES influence overweight and obesity. Regarding age, we used the same categories as other Spanish research groups investigating this topic [8,21,22]. This decision enables comparisons between studies and helps to establish a time trend, one of our primary aims.

In a previous study by Salcedo et al. performed in Spain [22], girls showed an increased prevalence of both overweight and obesity, while rates for boys peaked in 2001 and then trended toward normalization in 2006–2007. Another study in our country actually observed a net decrease in the prevalence of obesity in boys aged 5 to 9 years [23]. 

On the other hand, the upward trend in the prevalence of overweight and obesity between 1997 and 2007 is similar to that observed in different previous Spanish studies, while the downward trend between 2007 and 2017 is similar to a study conducted in a Spanish region (Catalonia), where weight and height were measured routinely measured by pediatricians or pediatric nurses in primary care centers to compare the prevalence of overweight and obesity between 2006 and 2016 [24].

Other research into trends in childhood BMI have reported heterogeneous results. While some studies in northern and central Europe [25,26] have observed a stabilization or even a decrease [4,27] in the prevalence of overweight and obesity, other investigations have pointed to an increase in children’s BMI [3].

These differences may be due to the age groups under study. Seven-year-olds in Hungary and 8–9-year-olds in Italy demonstrated stabilizing levels of overweight and obesity [28]. While other researchers have reported similar findings in preschool and primary school-aged children, these trends did not hold in older children or adolescents [23,24,29]. One possible explanation is that younger children are more susceptible to public health messages than pre-adolescents or adolescents, whose behavior patterns are more entrenched. A cohort effect or younger children’s lower exposure to risk factors would also explain the results [23].

In addition to the ages studied, there may be differences between countries: stabilization is clearer in northern compared to southern Europe or countries with economies in transition in eastern Europe, where rates are increasing [30]. One 2015 review in 25 countries showed an increase in prevalence of overweight in 13 countries, nine of which were in eastern Europe [30]. The divergence between countries in prevalence rates for overweight and obesity may occur at young ages: a comparison between Sweden and Spain showed that by the age of 4 years, children in Spain had already overtaken their Swedish counterparts in the prevalence of excess weight [31].

Regarding sex, our results indicate an increase in the prevalence of overweight and obesity in girls aged 10 to 15 years, along with stabilizing rates in those aged 5 to 9 years. These observations corroborate other studies in Spain reporting differential trends between boys and girls, where prevalence of high BMI showed a clearer tendency to stabilize or even decrease in boys compared to girls [8,22].

In Europe and in other high-income countries, these differences between genders are also apparent. Primary school-aged girls in Ireland and Australia, as well as girls in Poland and Estonia, all show higher prevalence of excess weight than their male counterparts [30,32]. On the other hand, some studies have observed the opposite: a stabilization in obesity trends in girls but not in boys [5], or even a decreasing prevalence of overweight in girls [33].

In light of the above, it seems reasonable to consider gender as a determinant of overweight and obesity and, by extension, of the effectiveness of interventions aimed at its prevention. If biological factors are undoubtedly essential in determining body weight, social and gender-related factors should also be considered with regard to food and diet, body image, physical activity, and sedentary behaviors [30].

In terms of the evolution over time, the link between risk of overweight in children and the SES of their parents emerges earlier in boys than in girls (1995–1997 data in boys and 2007 data in girls), but the strongest association is in girls in the last survey period (2017). For obesity, the risk associated with parents’ low educational level decreased in boys between 1995–1997 and 2017, but in girls, a significant association first appeared in 2017, with parents’ low educational level linked to double the risk of obesity compared to girls whose parents had a higher educational level.

Looking at the trends in excess weight according to socioeconomic differences, obesity rose in girls in 2017, reflecting a greater increase in prevalence among girls whose parents had a lower educational level. In contrast, between 2007 and 2017, socioeconomic differences in overweight for both boys and girls, and for obesity in boys, decreased, indicating that in these cases the decline in prevalence was greater in children whose parents had a low educational level.

In our study, we assessed SES as the educational level of the head of household, in line with previous research, showing that this measure is a more faithful indicator than occupation or income [11,34,35].

Different studies in high-income countries have reported a relationship between excess weight and family SES [4,36,37], while other authors have described an inverse gradient between overweight/obesity and SES, with BMI decreasing as SES increases [7,38]. Thus, in England, obesity increased among the most underserved populations from 2007–2008 to 2011–2012 [39]. This weight-related social inequality also increased in children aged 5–6 years [40].

There are also differences related to countries’ level of economic development. For example, one World Health Organization study in Europe found that low SES conferred a risk of overweight and obesity in Sweden, Portugal, and the Czech Republic, but in Bulgaria and Lithuania, the opposite occurred [41]. 

These differences may be due to differences between families with high versus low SES in access to healthy food [40], dietary habits [4,36,40], or sedentary behaviors [42], or different aesthetic perceptions about weight [8]. Some authors have even speculated that health promotion campaigns may disproportionately benefit people with higher SES [41]. Such findings have prompted calls for health promotion programs to go beyond the strict boundaries of individual, social, or environmental levels, adopting multifactorial approaches that encompass individual and environmental interventions [43] with a specific focus on the needs of marginalized groups.

Furthermore, the apparent turning point of the obesity epidemic in developed countries should be interpreted with caution. Indeed, in addition to the above considerations, the prevalence of morbid obesity continues to rise in these age groups [44] and shows an inverse relationship with the head of household’s educational level. The rising prevalence of obesity is also shifting from young adult age groups into younger cohorts [45].

### Study Limitations

This study uses a representative sample of children in Spain aged 5 to 15 years. We excluded children with missing data for weight or height, which may have increased the risk of information bias if the participants with missing data were systematically different from those without.

We used the weights and heights reported by the head of household, a measure that has shown reasonable validity for assessing obesity in epidemiological studies [16,18,20]. We must assume some risk of measurement bias, as parents may tend to underestimate their children’s weight. However, the data collection method was the same in all three surveys, so we believe that the comparison is valid.

We have used the cut-off points for overweight and obesity recommended by the International Obesity Task Force [14]. However, we cannot rule out that the results were different with other cut-off points. However, we have analyzed the BMI as a quantitative variable and the magnitude of the mean obtained shows a trend similar to that observed with overweight and obesity.

## 5. Conclusions

The upward trend in prevalence of overweight and obesity between 1997 and 2007 reversed direction from 2007 to 2017, showing a modest downtick. The exception was in girls aged 10 to 15 years, in whom prevalence of overweight and obesity rose during the last 10 years of the study period. From 2007 to 2017, the decrease in prevalence of overweight in both sexes and of obesity in boys, along with the increase in prevalence of obesity in girls, was of higher magnitude in children whose parents had a lower educational level. The apparent turnaround in the obesity epidemic in Spain should be interpreted with caution. Children’s body weight is influenced by both gender and socioeconomic status—considerations that should be kept in mind when designing health promotion interventions.

## Figures and Tables

**Table 1:** Prevalence (95% confidence interval) of overweight and obesity in boys and girls aged 5 to 9 years. Spain, 1997, 2007, and 2017.

Variables	1997	2007	2017	*p*
**Boys**				
Number	377	894	889	
Overweight, P(95%CI)	34.5 (29.7–39.3)	33.4 (30.4–36.3)	30.8 (27.9–33.7)	0.346
Obesity, P(95%CI)	14.9 (11.3–18.4)	14.4 (12.2–16.6)	13.2 (11.5–15.3)	0.477
**Girls**				
N	323	883	783	
Overweight, P(95%CI)	35.0 (29.8–40.2)	37.2 (34.2–40.3)	35.5 (32.4–38.6)	0.302
Obesity, P(95%CI)	12.4 (8.8–16.0)	14.5 (12.3–16.7)	14.1 (11.9–16.4)	0.242

*p*: value for the statistical significance for time trend from 1997 to 2017. CI—confidence interval.

**Table 2:** Prevalence (95% confidence interval) of overweight and obesity in boys and girls aged 10 to 15 years. Spain, 1997, 2007, and 2017.

Variables	1997	2007	2017	*p*
**Boys**				
Number	664	1561	1091	
Overweight, P(95%CI)	21.2 (18.1–24.3)	27.0 (24.7–29.2)	24.8 (22.1–27.5)	0.059
Obesity, P(95%CI)	3.6 (2.2–5.0)	5.2 (4.1–6.3)	4.9 (3.6–6.3)	0.167
**Girls**				
N	651	1478	1113	
Overweight, P(95%CI)	14.6 (11.9–17.3)	17.7 (15.7–19.7)	19.6 (17.1–22.2)	0.143
Obesity, P(95%CI)	1.1 (0.3–1.9)	3.2 (2.3–4.1)	3.7 (2.5–4.9)	0.298

*p* for time trend from 1997 to 2017. CI—confidence interval.

**Table 3:** Association between parents’ educational level and children’s risk of overweight and obesity. Spain, 1997, 2007, and 2017.

Varaibles	Odds Ratio (95% Confidence Interval)					
	1997 (1783)		2007 (4616)		2017 (3570)	
Overweight						
**Boys**						
High education	1.00		1.00		1.00	
Low education	1.39	(1.04–1.87)	1.43	(1.20–1.70)	1.40	(1.12–1.71)
**Girls**						
High education	1.00		1.00		1.00	
Low education	1.27	(0.91–1.76)	1.69	(1.38–2.05)	1.58	(1.28–1.94)
Obesity						
**Boys**						
High education	1.00		1.00		1.00	
Low education	1.65	(0.99–2.74)	1.56	(1.17–2.08)	1.31	(0.96–1.78)
**Girls**						
High education	1.00		1.00		1.00	
Low education	0.85	(0.46–1.58)	1.28	(0.93–1.76)	2.03	(1.46–2.83)

## Data Availability

Publicly available datasets were analyzed in this study. This data can be found here: https://www.mscbs.gob.es/estadisticas/microdatos.do.

## Supplementary Materials

The following are available online at https://www.mdpi.com/1660-4601/18/4/1842/s1. Figure S1. Mean body mass index in boys aged 5 to 9 and 10 to 15 years. Figure S2: Mean body mass index in girls aged 5 to 9 and 10 to 15 years.
