# Supplementary material for: Reversal of the Upward Trend of Obesity in Boys, but Not in Girls, in Spain
Source: Int J Environ Res Public Health. 2021 Feb 14;18(4):1842. doi: 10.3390/ijerph18041842 (PMC7918759; doi:10.3390/ijerph18041842)

Figure S1. Mean body mass index in boys aged 5 to 9 and 10 to 15 years

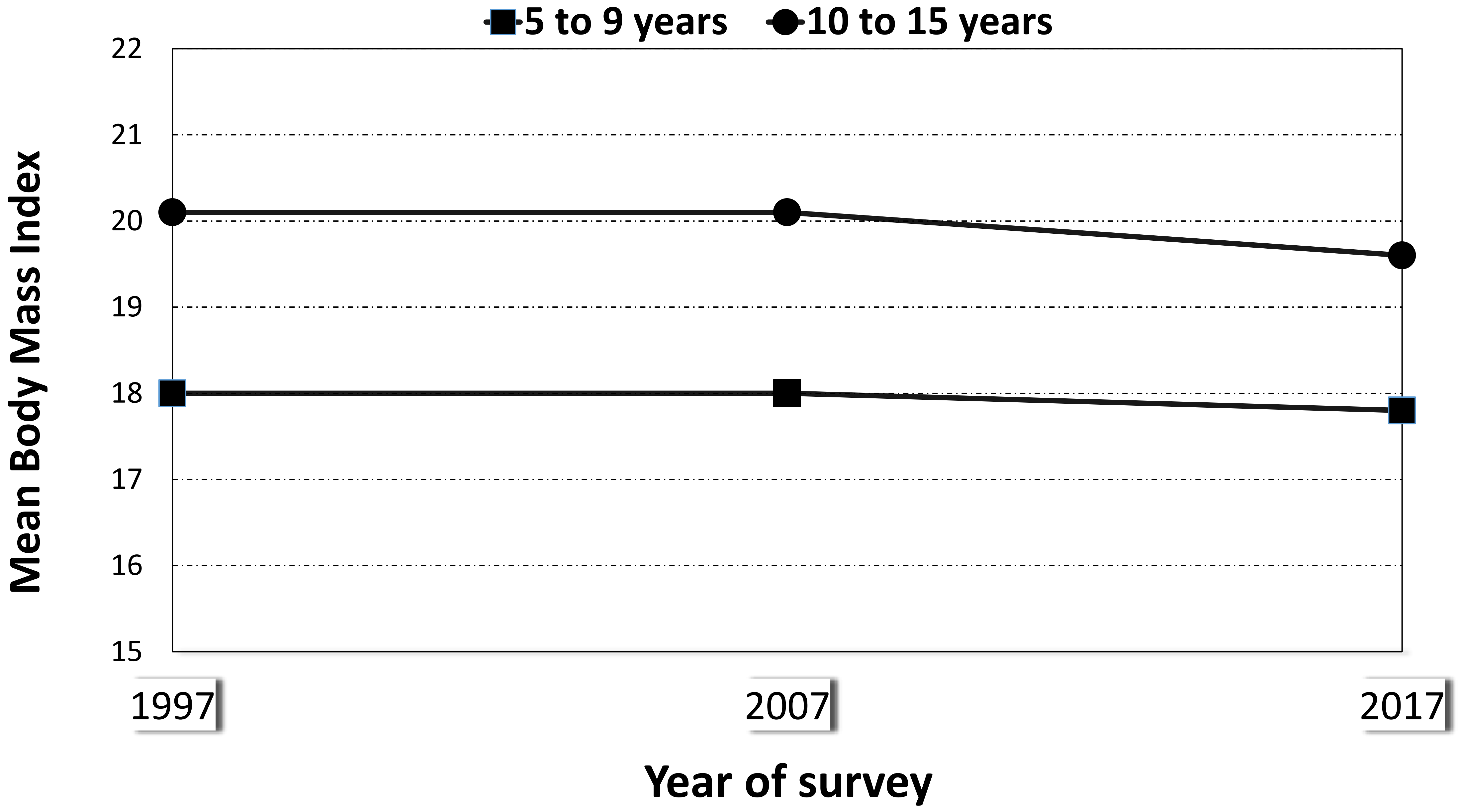

Figure S2. Mean body mass index in girls aged 5 to 9 and 10 to 15 years

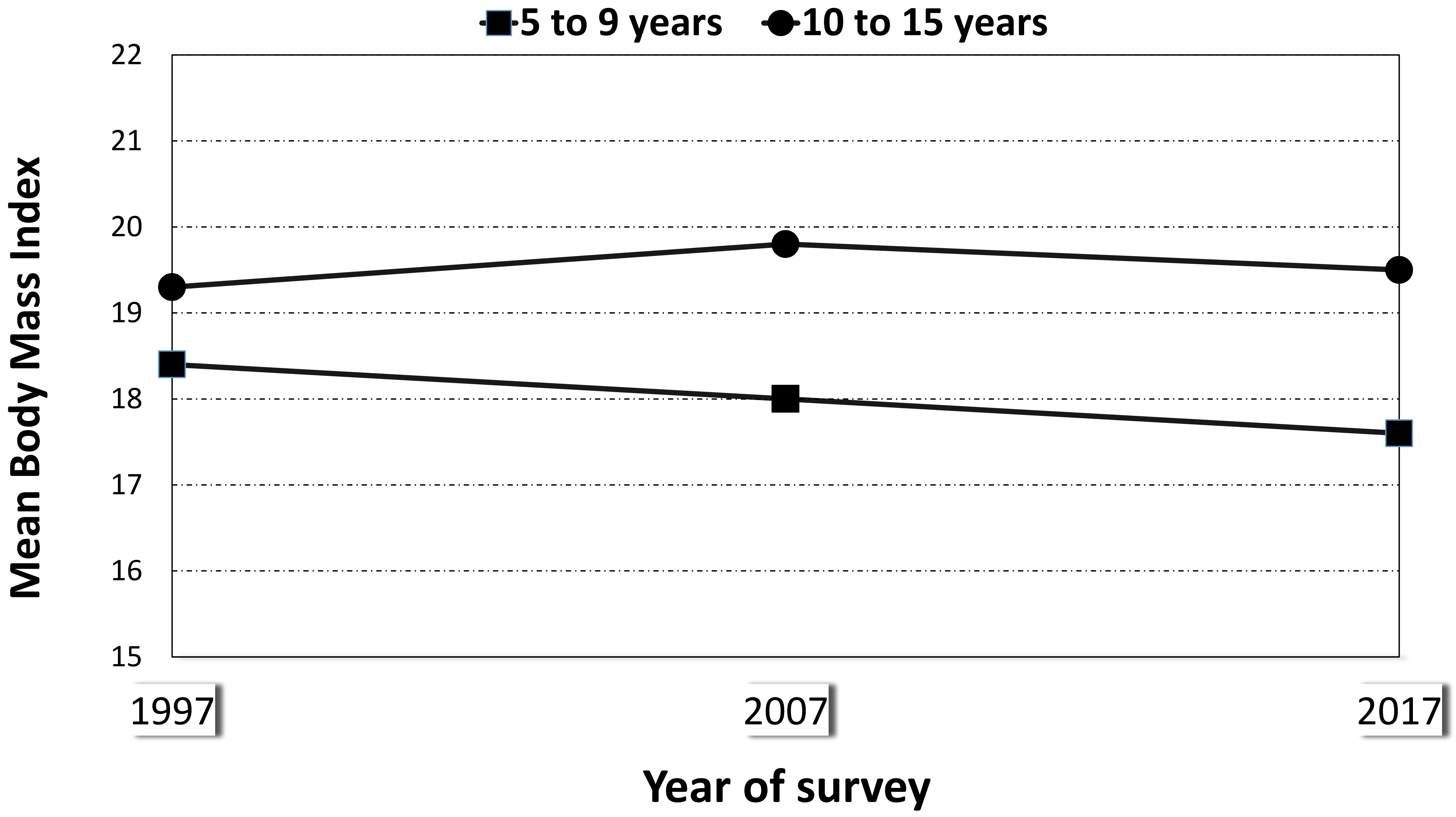

Supplement: Supplementary file 1 [file ijerph-18-01842-s001.pdf]
